# High Performance Colored Solar Absorber Coatings for Architectural Value

**DOI:** 10.3390/ma19040703

**Published:** 2026-02-12

**Authors:** Hsuan Chu Lai, Fu-Der Lai, Ching-Wen Cheng, Yen-Ting Lai, Jian-Yu Tong

**Affiliations:** 1 Graduate School of Business and Leadership, National Louis University, Tampa, FL 33634, USA; 2 Institute of Photonics Engineering, National Kaohsiung University of Science and Technology, Kaohsiung 807618, Taiwan; 3 Department of Information and Computer Engineering, Chung Yuan Christian University, Taoyuan City 320314, Taiwan; jeremy10350206@gmail.com

**Keywords:** surface roughness, self-cleaning capability, service lifetime, viewing angle, environmental durability, architectural application, high brightness

## Abstract

Solar absorbers (SAs) are central to building-integrated solar-thermal systems; however, conventional black SAs, despite their high solar absorptance (α_s_), offer limited aesthetic flexibility and are therefore poorly suited to modern architectural façades. Brightly colored SAs are widely assumed to suffer from intrinsically low α_s_, creating a long-standing trade-off between color vibrancy and energy performance. Here this study reports a dielectric/absorber/dielectric/absorber (D/A/D/A) multilayer architecture, in which the absorber layer is composed of a TiO_2_–TiON–C composite, that overcomes this limitation and enables colored solar absorbers (CSAs) with reflectance >20%, α_s_ > 0.90, wide viewing angles, strong self-cleaning capability, high corrosion resistance and exceptionally long projected service lifetimes. These results demonstrate that vivid coloration and high solar absorptance can be simultaneously achieved without compromising environmental durability. To highlight architectural applicability, we further implement a complementary-color contrast strategy for façade design, yielding visually striking, highly recognizable, and low-cost exterior surfaces. This approach enhances aesthetic integration while significantly strengthening the marketability of CSA-based building envelopes for next-generation sustainable architectural systems.

## 1. Introduction

Improving energy efficiency in buildings is a crucial component of global sustainability efforts and is closely aligned with Sustainable Development Goal 7, which prioritizes access to affordable and clean energy. Among various strategies, integrating solar-thermal technologies into architectural systems has emerged as an effective approach for reducing building energy consumption. Solar air-heating configurations, for instance, can be incorporated into both new constructions and retrofitted structures to provide passive space heating or ventilation [1]. Large-area solar selective absorbers (SSAs) are particularly well suited for such applications and can be installed on rooftop structures, façade panels, or vertical ventilation shafts, where they convert incident sunlight into thermal energy. The resulting buoyancy-driven airflow enhances natural ventilation by drawing cooler air from lower floors or outdoor inlets. A representative implementation is the solar-chimney-assisted natural ventilation system at the University of Kitakyushu, Japan, where solar chimneys combined with underground cooling pits significantly reduce reliance on mechanical air conditioning [2]. Beyond ventilation, façade-integrated SSAs can further supply heat for domestic hot water, space heating, and low-temperature industrial processes [3,4].

Due to their simple design, low fabrication cost, high solar absorptance (α), and low thermal emittance (ε), Dielectric–Absorber (D–A) multilayer solar selective absorbers (SSAs) have been extensively investigated. In these structures, the absorber layer may consist of a metal, an absorbing dielectric, or a composite material, among which metals (M) are the most commonly employed. Dielectric–Metal–Dielectric (DMD)-structured SSAs are widely studied due to their simple design, low cost, high α_s_, and low ε [5]. By adjusting the thicknesses of D and M layers, DMD structures can be engineered to exhibit various colors [5,6,7], enabling their integration into architecturally exposed components. However, for successful architectural integration, the color and intensity of reflected light from Colored Solar Absorbers (CSAs) should remain invariant with changes in viewing angle. Angular-dependent variations in appearance compromise the intended design aesthetics, creating visual mismatches between CSA coatings and building façades, potentially rendering them unacceptable to users with high aesthetic expectations. Nevertheless, current multilayer CSA coatings with flat surfaces often exhibit noticeable color and brightness changes at oblique viewing angles, which limit their widespread adoption. Therefore, solving the issue of angular-dependent optical variation is essential for promoting CSA coatings in architectural applications.

DMD-based absorbers are considered promising candidates for developing high-performance CSAs [5,6,7,8] and have been extensively studied. Z.Y. Nuru et al. have reported on Al_x_O_γ_/Pt/Al_x_O_γ_ structures [6,8,9], including their optimization, structural and optical properties, thermal stability, and heavy ion elastic recoil detection analysis. Other Al_x_O_y_/M/Al_x_O_y_ absorbers have also been explored, such as Al_2_O_3_/Ti/Al_2_O_3_ [10], Al_2_O_3_/Mo/Al_2_O_3_ [11], Al_x_O_y_/Cr/Al_x_O_y_ [12], Al_x_O_y_/Al/Al_x_O_y_ [13], Al_2_O_3_/M/Al_2_O_3_ (M—Ni, Cr, Ta, Pt and Mo) [14], and Al_x_O_y_/Ni/Al_x_O_y_ [15]. Similarly, SiO_2_/M/SiO_2_ multilayer absorbers using Ti [16], Cr [17], and W [18] have been studied. Cr_x_O_y_/Cr/Cr_x_O_y_ structures have been investigated for their structural and optical properties via pulsed sputtering [19], as well as their growth characteristics [20], optical simulation, and thermal stability [21]. MgO/Zr/MgO multilayer coatings have been analyzed for their optical properties, thermal stability [22], and annealing-induced interdiffusion [23]. High-temperature HfO_x_/Mo/HfO_2_ SSAs deposited by pulsed sputtering have also been reported by N. Selvakumar et al. [24]. Additionally, alternative multi-layer configurations similar to DMD, such as DDMD (SiO_2_/Si_3_N_4_/W/SiO_2_) [25] and DMDD (ZrO_x_/Zr/ZrO_x_/Al_x_O_y_ [26] or AlN/Ti/AlN/SiO_2_ [27]), have been investigated. A comprehensive review of spectrally selective dielectric–metal–dielectric coatings has been provided by A. Dan et al.

However, despite their functional advantages, black SSAs are generally unsuitable for integration into exterior façades or solar chimney systems, particularly in modern architectural contexts where aesthetic considerations are paramount. Although high-brightness CSAs are far more desirable from a design standpoint, their adoption in building envelopes remains limited due to two major barriers: high fabrication cost and several long-standing misconceptions. It is widely believed that vividly colored coatings inevitably exhibit low α_s_, suffer from narrow viewing angles with pronounced angular-dependent color and brightness variations, and are prone to surface contamination, making them impractical for large-area architectural deployment. These challenges collectively highlight the need for a new class of solar-thermal coatings with two essential characteristics. First, the material must achieve a combination of high brightness, high α_s_, wide viewing-angle stability, strong self-cleaning capability, and long operational lifetime, thereby uniting architectural aesthetics with robust solar-thermal performance and long-term environmental durability. We introduce a dielectric/absorber/dielectric/absorber (D/A/D/A) multilayer architecture that overcomes the traditional trade-off between color and performance in solar absorbers. The absorber layers, composed of a TiO_2_–TiON–C composite, enable D/A/D/A structured CSAs that simultaneously achieve a high CIE Y value (reflectance > 20%), an α_s_ exceeding 0.85, and a wide viewing angle. Furthermore, these devices exhibit robust self-cleaning capabilities and an exceptionally long service life. This study demonstrates that vivid coloration, high-efficiency solar-thermal conversion, and outstanding environmental durability can be successfully integrated. Secondly, practical building applications require low-cost manufacturing processes; therefore, this study further examines the practical building applications underlying complementary-color façade design and presents three representative examples that demonstrate the significant application potential of CSAs for next-generation building-integrated solar-thermal applications.

## 2. Experimental Details

### 2.1. Preparation of the D/A/D/A CSA Coatings

Based on thin-film optics theory, achieving both high brightness and high solar absorptance necessitates an absorber layer with a low extinction coefficient (*k*) and a refractive index (*n*) close to that of Al_2_O_3_. To tailor the refractive index accordingly (given that carbon exhibits an *n* < 1.4), carbon was incorporated into the composite. Therefore, the absorber (A) is formulated as a TiO_2_–TiON–C composite, while the dielectric layer consists of Al_2_O_3_.

**Figure 1:**
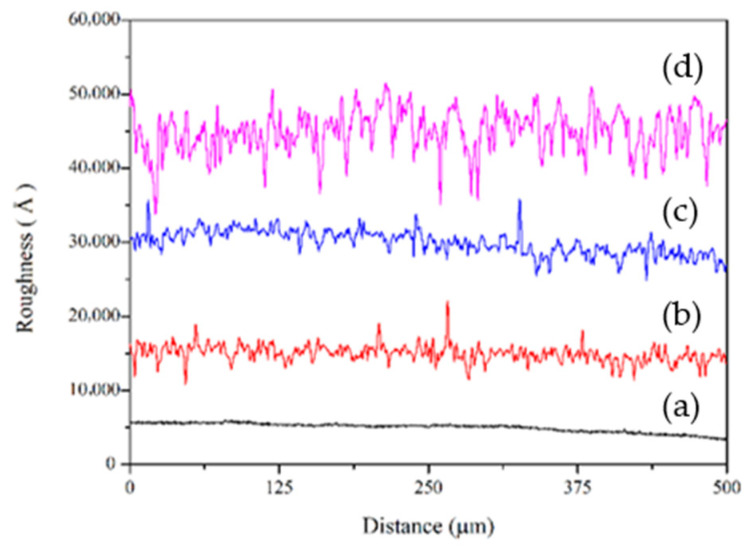
The surface morphologies for (**a**) EP, (**b**) #1500, (**c**) #1000, and (**d**) #600.

An unbalanced reactive magnetron sputtering system with a cryo pump is used to prepare the D/A/D/A multilayer CSA coatings. Mirror-like SS304 stainless steel with a root mean square (RMS) surface roughness of approximately 100 nm is achieved through electrolytic polishing. TiO_2_–TiON–C absorber layers with a nominal thickness of ~200 nm were deposited onto SS304 stainless steel substrates that had been preconditioned to different levels of surface roughness. The substrates were first finished either by Electrolytic Polishing (EP) (root-mean-square roughness Ra ≈ 50 nm) or by mechanical abrasion using #1500 (≈Ra 80 nm), #1000 (≈Ra 120 nm), and #600 (≈Ra 200 nm) grit sandpapers [7,16]. Their morphologies, measured by α steper (KLA Tencor, Alpha-Step IQ), are shown in Figure 1. Subsequently, dielectric/absorbing/dielectric/absorbing (D/A/D/A) multilayer CSA coatings—designated as C-EP, C#1500, C#1000, and C#600—were deposited onto pretreated SS304 substrates. The target materials for the Al_2_O_3_ and Ti are aluminum (99.995%) and titanium (99.995%), respectively. To eliminate any Al_x_O_1−x_ and Ti contaminants, the target was pre-sputtered in an argon atmosphere at a total pressure of 20 mTorr for 20 min. Following this step, D/A/D/A multilayer coatings were deposited using the specified deposition conditions. The Al_2_O_3_ film is deposited at 3 mTorr total pressure, 30 sccm Ar flow rate and 10 sccm O_2_ flow rate, 300W RF sputtering power from an Al target. The TiO_2_–TiON–C composite layers are deposited via DC pulse magnetron sputtering under a total pressure of 3 mTorr, using the gas flow rate 2 (O_2_), 10 (N_2_), and 10 (C_2_H_2_) sccm and a sputtering power of 180 W applied to the titanium target.

### 2.2. Characterization of the CSA Coatings

With the integrating sphere (ISN-723), a high-performance double-beam UV-Visible-NIR spectrophotometer (JASCO V-670) is used to measure the reflectance spectrum in the wavelength ranges of 250–1900 nm. The refractive index *n* and extinction coefficient *k* of the Al_2_O_3_ and Ti films are measured by the spectroscopic ellipsometer (J. A. Woollam, M-2000 DI). All color photos are taken by a Canon camera under the sun’s rays. The crystallographic structures of the CSA coatings are measured by Grazing Incidence X-ray diffraction (Bruker, D8 DISCOVER with GADDS). Energy-dispersive X-ray spectroscopy (EDS) analysis (ULVAC-PHI, PHI VersaProbe 4) determined the elemental composition of the absorber layer as 60.35% TiO_2_, 7.65% TiON, and 32.0% C, which was further confirmed by SEM-EDS results (Hitachi, SU-5000). For electrochemical potentiodynamic measurement (CH Instruments, CHI6273E), the experimental arrangement used a three-electrode system. Scans were initiated by lowering the corrosion potential of the sample from a preset value of −1.0 V (vs. SCE) to 0 V at a rate of 0.5 mVs^−1^. All experiments were conducted in 1N NaCl solution at a room temperature of about 25 °C. To assess the adhesion of the coating, Vickers indentation tests were conducted under a compressive load of 100 g, and the crack propagation and delamination behavior around the indentations were examined. To evaluate the self-cleaning potential of the surface, the water contact angle measurements were employed.

### 2.3. Analysis of Solar Absorptance (α_s_) for the Al_2_O_3_/Ti/Al_2_O_3_ CSSAs

α_s_ is obtained by weighting the measured reflectance spectrum with the spectral power distribution of the standard AM 1.5 solar spectrum and performing spectral integration, as described in Ref. [28].

(1) $$\alpha =1-\frac{\int_{0.25}^{2.0}R(\lambda )\cdot SD(\lambda )d\lambda }{\int_{0.25}^{2.0}SD(\lambda )d\lambda }$$

where R(λ) and SD(λ) are, respectively, the measured reflectance and the spectral power density of AM1.5 at the wavelength of λ.

## 3. Results and Discussion

### 3.1. Optical Characteristics of High-Brightness and High-Solar-Absorptance Coatings

#### 3.1.1. Design of Optical Constant of Absorber Layer for High-Brightness and High-Solar-Absorptance

Based on thin-film optical theory, the simultaneous realization of high brightness and high solar absorptance requires an absorber layer with a low extinction coefficient (k) and a refractive index (n) close to that of Al_2_O_3_. To achieve appropriate refractive-index matching, carbon, whose refractive index is lower than 1.4, was incorporated into the absorber material to tailor its effective optical constants. Accordingly, the absorber layer was designed as a TiO_2_–TiON–C composite, while the dielectric layer consisted of Al_2_O_3_. Furthermore, in the D/A/D/A multilayer architecture, both absorber layers (the second and fourth layers) actively contribute to solar-energy absorption. In particular, the fourth absorber layer, with a relatively large thickness of approximately 250 nm, is responsible for absorbing the majority of the incident solar energy. This design strategy prevents the solar-energy absorption from relying solely on the much thinner second absorber layer, thereby enhancing overall absorption efficiency without compromising optical brightness.

#### 3.1.2. Debunking the Perception That “High-Brightness CSA Coatings Must Exhibit Low Solar Absorptance”

**Figure 2:**
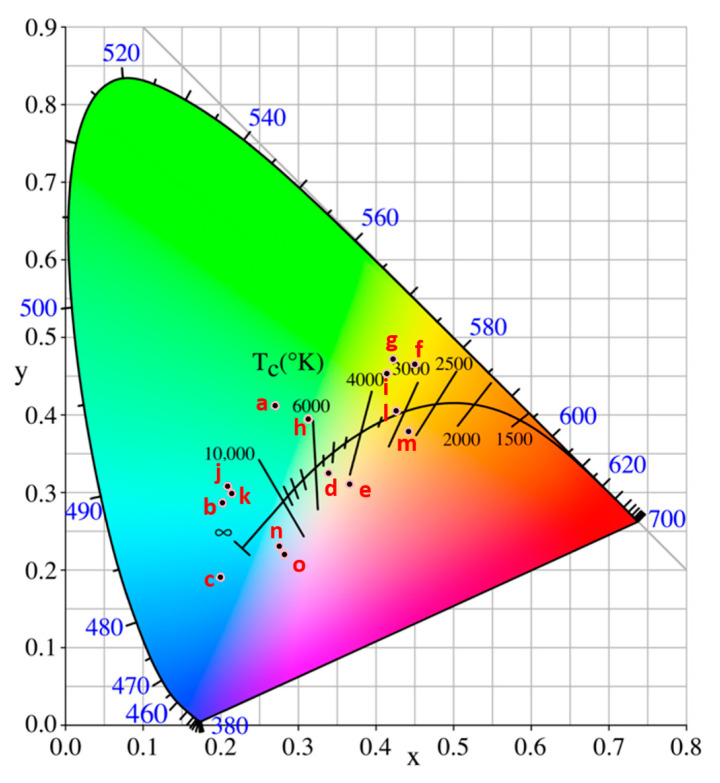
The distribution of 15 high-brightness and high-solar-absorptance CSA coatings across different color regions in the CIE xy chromaticity diagram. a–o are the test piece numbers as shown in Table 1.

**Figure 3:**
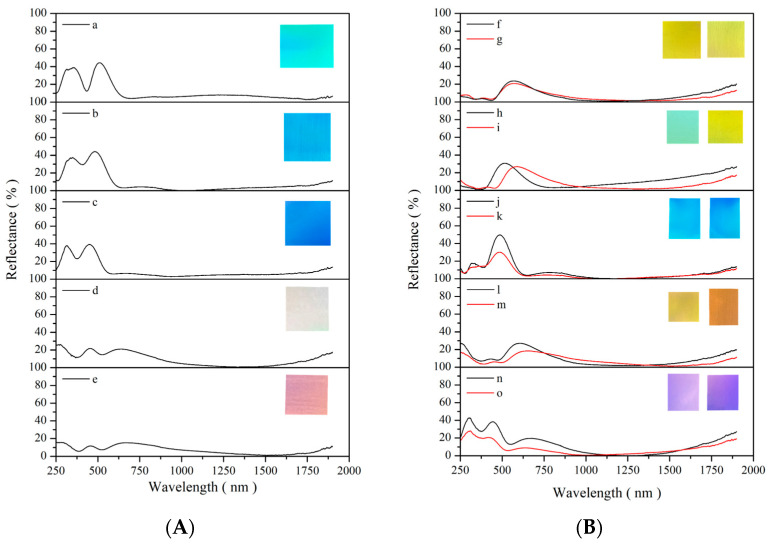
(A) Reflectance spectra and photographic images of the samples shown in Figure 2. (B) Reflection spectra and photographic images of the samples from Figure 1, simultaneously coated on both specular and rough surfaces. The test pieces are shown in Table 1. a–o are the test piece numbers as shown in Table 1.

Figure 2 shows the distribution of 15 high-brightness and high-solar-absorptance CSA coatings across different color regions in the CIE xy chromaticity diagram. Table 1 presents the corresponding high-brightness CSA samples (labeled according to Figure 2), all of which exhibit high visible reflectance—maximum reflectance (R_max_) values exceeding 15% and, in some cases, greater than 40%—while maintaining high α_s_. Within the visible wavelength range, the R_max_ of all samples exceeds 20%, except for samples e and m, whose R_max_ remains below 20%. As an example, the yellow-colored CSA coating (sample g) deposited on the #600 substrate exhibits an α_s_ of 90.4%, accompanied by a R_max_ of 20.9%. These results clearly demonstrate that CSA coatings can achieve both high brightness and high solar absorptance, disproving the common misconception that a high-brightness appearance inherently compromises solar-thermal performance. Figure 3 presents the reflectance spectra and photographic images of the specimens shown in Figure 2. Table 1 summarizes the corresponding surface conditions, α, R_max_ in the visible color region, and the associated characteristic wavelengths of the specimens in Figure 3.

### 3.2. Influence of Surface Roughness on the Optical Properties of CSA Coatings

#### 3.2.1. Effects on Solar Absorptance and Brightness

As shown in Figure 3B (depicting the same sample as in Figure 2 and Table 1), the optical performance of CSA coatings is significantly influenced by substrate surface roughness. The roughness values (peak-to-peak and Ra) for samples CEP, C#1500, C#1000, and C#600 were 64.4/19.8 nm, 86.8/19.3 nm, 353.6/43.0 nm, and 1435.3/242.2 nm, respectively. Increasing the surface roughness raised the α by an average of about 2% and concurrently decreased the maximum reflectivity by 7–20%. Nevertheless, all maximum reflectivity values were greater than 18.7% and the coatings maintained their high-brightness characteristics.

#### 3.2.2. Effects on Viewing Angle Range

The samples were first exposed to natural sunlight and visually examined to confirm stable and representative color and brightness. Photographs were then taken with a Canon digital camera only after the camera-rendered appearance was verified to be consistent with direct observation, thereby minimizing deviations caused by camera response or post-processing.

**Figure 4:**
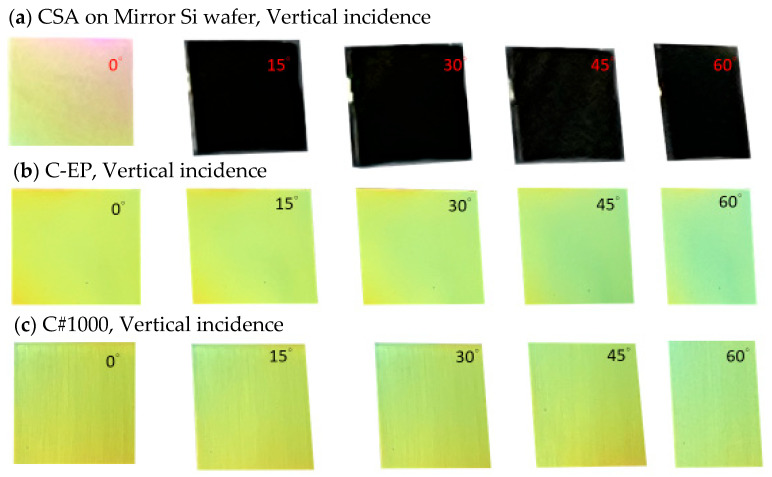
Comparison of viewing-angle-dependent appearances (0–60°) for coatings with different surface roughnesses. CSA coatings are deposited on (**a**) a mirror Si wafer, (**b**) an electropolished substrate, and (**c**) a rough substrate, published with #1000 sandpaper.

**Figure 5:**
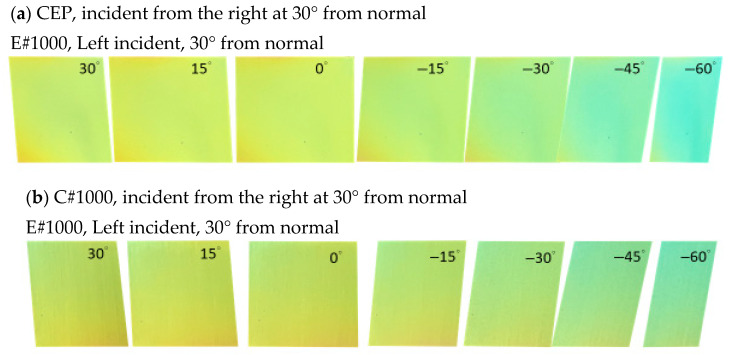
Comparison of angle-dependent photographic images at a −30° incident angle. Images show the appearance of (**a**) CEP and (**b**) C#1000 coatings.

All photographs were taken outdoors on the campus of the National Kaohsiung University of Science and Technology in November. Imaging was conducted under natural illumination approximately one to two hours before sunset, with ambient light intensity ranging from about 6000 to 20,000 lux. To investigate the influence of surface roughness on viewing-angle-dependent appearance, the samples were oriented nearly perpendicular to the incident sunlight direction (incident angle ≈ 3°), and images were captured at viewing angles of 0°, 15°, 30°, 45°, and 60°, as illustrated in Figure 4. As shown in Figure 4a, when sunlight impinges on the CSA coating deposited on a mirror-like silicon substrate, a bright and distinct color is observed only at a 0° viewing angle. At larger viewing angles, the appearance becomes dark due to specular reflection governed by Snell’s law, where the incident and reflection angles coincide. In contrast, the CEP coating (Figure 4b) deposited on the electropolished (EP) substrate exhibits a characteristic yellow appearance within the 0–30° viewing-angle range. At viewing angles exceeding 45°, a gradual blue–green tint emerges, becoming more pronounced with increasing angle. Notably, the C#1000 coating retains a yellow-dominant appearance with only slight blue–green components up to viewing angles approaching 60°, and remains visually yellow even at a 60° viewing angle. These observations indicate that increasing surface roughness effectively broadens the viewing-angle range over which the characteristic color is preserved. A second observation configuration was employed to further examine angular appearance behavior. In this setup, sunlight was incident at −30° relative to the surface normal and propagated from left to right. In other words, 30° is the angle at which sunlight is reflected from the surface of the mirror sample. Images were subsequently captured at viewing angles of 30°, 15°, 0°, −15°, −30°, −45°, and −60°, as shown in Figure 5. Since the reflected beam propagates along the 30° direction, viewing angles spanning from 75° to 30° deviate from the reflected light direction by only 45°. This geometric alignment preserves high reflected intensity, resulting in a yellow appearance. As shown in Figure 5a, the C-EP-coated sample maintains a bright yellow appearance from 75° to −15°. At −30°, the yellow color persists with a slight blue–green tint, while at more oblique angles, the color progressively shifts toward blue–green, becoming dominant at −60°. In comparison, the C#1000 coating (Figure 5b) exhibits a significantly wider viewing-angle tolerance, maintaining a bright yellow appearance from 75° to −30°. Even at −45°, the coating retains its characteristic yellow hue, with only a mild blue–green contribution, and at −60°, the appearance remains yellow-dominant.

Although this study did not perform angle-dependent quantitative analysis, it is evident that increased surface roughness could widen the effective viewing angle range.

### 3.3. Environmental Durability Analysis of High Brightness CSAs

The durability analysis of the high-brightness CSA environment includes tests such as adhesion analysis, self-cleaning ability, thermal aging test, and weather resistance assessment through a corrosion test.

#### 3.3.1. Adhesion Assessment and Analysis Using the Vickers Indentation Method

Table 2 shows the Vickers micro-indentation results under a 100 g load. The CEP sample corresponding to Image 1 showed well-defined indentation edges without delamination, suggesting good adhesion strength. The better performance of the C#1000 sample was observed in Image 2, where the coating accommodated the indentation without crack formation or peeling, demonstrating excellent toughness and the strongest interfacial adhesion among all samples. These results indicate that surface conditions impact adhesion behavior, with substrate roughness enabling strong mechanical interlocking and superior resistance to crack propagation.

#### 3.3.2. Impact of Surface Roughness on the Self-Cleaning Potential of the Coatings

To evaluate the self-cleaning performance of the CSA coatings, the water contact angles were measured by analyzing side-view droplet images on the coating surfaces. A tangent line was drawn at the three-phase (solid–liquid–air) contact point to determine the wetting behavior and infer the contaminant removal capability. Table 3 lists the water contact angles of CSA coatings deposited on substrates with different surface roughnesses: 92° for CEP, 95° for C#1500, 108° for C#1000, and 103° for C#600. All contact angles exceed 90°, indicating that every sample exhibits hydrophobic behavior. Compared with the CEP sample, all coatings on roughened surfaces show enhanced self-cleaning performance. However, the results also indicate that a rougher surface does not necessarily lead to a larger contact angle. Although the C#600 substrate is rougher than C#1000, its contact angle is lower. The C#1000 sample exhibits the largest contact angle (108°), demonstrating the strongest hydrophobicity and thus the most effective self-cleaning behavior via the rolling-droplet mechanism. Overall, the Al_2_O_3_/TiO_2_–TiON–C/Al_2_O_3_/TiO_2_–TiON–C multilayer coatings exhibit excellent self-cleaning properties, with the C#1000 surface providing the best performance among all tested substrates.

This study develops CSA coatings with water contact angles exceeding 90°, indicating hydrophobic behavior, for applications on building façades or highly inclined ventilated roofs. Under such conditions, water droplets can readily roll off the coating surface. This rolling effect provides a self-cleaning function, effectively reducing the accumulation of dust and contaminants, and consequently significantly decreasing the labor and time required for routine cleaning and maintenance.

#### 3.3.3. Annealing-Induced Degradation and Structural/Optical Evolution Analyses

To assess the impact of annealing temperature on the accelerated aging behavior of CSA coatings, X-ray diffraction (XRD), and optical reflectance spectra were employed. Lifetime is also estimated.

##### Composition and Phase Evolution Analyzed by XPS and XRD Under Thermal Annealing

**Figure 6:**
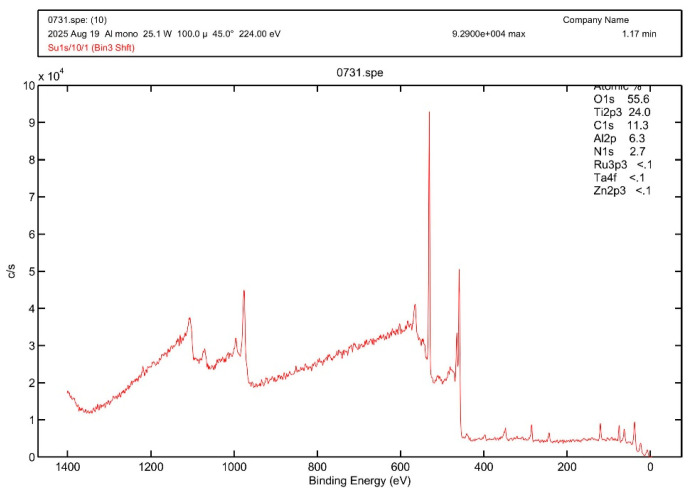
XPS compositional analysis of the absorber layer. The results reveal the presence of TiO_2_ (60.35%), TiON (7.65%), and C (32%) in the layer sputtered on the Al_2_O_3_/Ti/SS304 substrate.

When Ti was sputtered on the Al_2_O_3_/Ti/SS304 substrate in a mixed atmosphere of O_2_, N_2_, and C_2_H_2_, XPS compositional analysis, as shown in Figure 6 of the resulting absorber layer, revealed TiO_2_, TiON, and C with mole fractions of 60.35%, 7.65%, and 32%, respectively. In an O_2_/N_2_/C_2_H_2_ mixed reactive sputtering atmosphere, the strong affinity between Ti and oxygen favors TiO_2_ formation, while limited oxygen availability leads to the coexistence of TiON. Carbon introduced from C_2_H_2_ competes with oxygen and nitrogen, resulting in excess free carbon when the carbon flux exceeds Ti bonding capacity.

**Figure 7:**
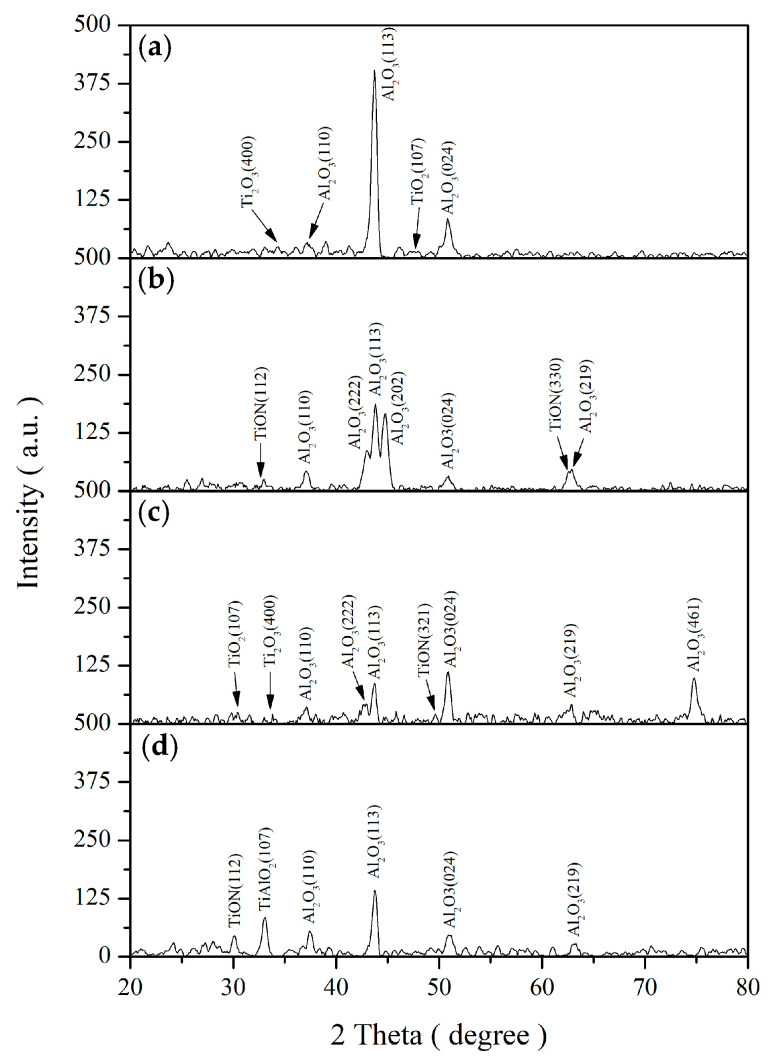
XRD patterns of the CSA coatings at (**a**) room temperature and after annealing at (**a**) 25 ℃, (**b**) 350 °C, (**c**) 450 °C, and (**d**) 550 °C for 12 h.

Figure 7 presents the XRD patterns of the CSA coatings after heat annealing at room temperature, 350, 450, and 550 °C for 12 h. Distinct crystalline phases of Al_2_O_3_, TiO_2_, and TiON are observed at 350 and 450 °C. The diffraction peaks can be clearly indexed to crystalline TiO_2_ and Al_2_O_3_, accompanied by weak reflections associated with a TiN_x_O_γ_ (TiON-like) phase, indicating partial oxidation of the nitride-related component while preserving the overall structural integrity of the coating. Annealing at 550 °C, the diffraction peaks corresponding to TiO_2_ and Al_2_O_3_ become more pronounced, and additional reflections attributable to TiAlO_2_ are clearly identified, indicating the formation of a titanium–aluminum mixed oxide phase through solid-state reaction between TiO_2_ and Al_2_O_3_ at elevated temperatures [16]. Such structural evolution is consistent with the observed optical behavior, where moderate-temperature annealing preserves high α, while prolonged exposure at higher temperatures leads to accelerated optical degradation.

Notably, no TiC or crystalline carbon phases are detected, despite the presence of carbon (32%) confirmed by XPS. These findings lead to two conclusions:

(1) Ti does not react with C to form TiC during sputtering in O_2_–N_2_–C_2_H_2_ atmospheres; and
(2) The carbon incorporated in the absorber layer exists in a dispersed, non-crystalline state rather than forming aggregated or carbide phases.

##### Effect of Annealing Temperatures on Optical Reflectance and Degradation Rate of CSA Coatings

**Figure 8:**
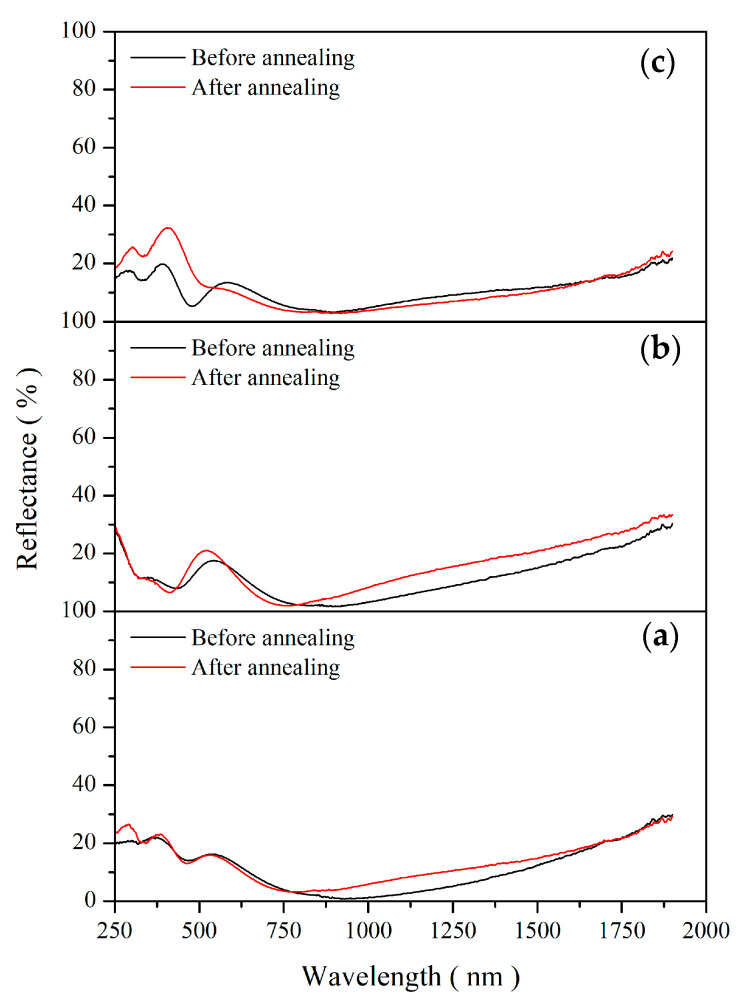
Optical reflectance spectra of the samples before and after thermal annealing at (**a**) 350, (**b**) 450, and (**c**) 550 °C.

Figure 8 presents the optical reflectance spectra of the samples before and after thermal annealing at 350, 450, and 550 °C. Table 4 summarizes the annealing temperatures, α before and after annealing, and the calculated degradation rates for the specimens shown in Figure 8.

As shown in Figure 8 and Table 4, all as-deposited samples exhibit high solar absorptance (α_s_ > 91%). After annealing at 350, 450, and 550 °C, the corresponding degradation rates are 0.70%, 1.77%, and 1.06%, respectively, indicating excellent thermal stability within this moderate-temperature range. Notably, the degradation at 550 °C is slightly lower than that at 450 °C, suggesting a non-monotonic thermal response. XRD analysis reveals that annealing within this temperature range promotes the crystallization and phase evolution of TiO_2_ and Al_2_O_3_, while also maintaining the persistence of TiON-related phases. The formation and stabilization of these oxide phases are believed to enhance the structural integrity of the coating, thereby mitigating optical degradation at elevated temperatures. Based on the activation energies of the constituent phases—TiO_2_ (2.0 eV, 60.35%), TiON (1.9 eV, 7.65%), and C (0.7 eV, 32.00%)—the Arrhenius-based lifetime estimation predicts that the service lifetime of the CSA coatings at 400 K exceeds 300 years, where failure is defined as a 30% reduction in α. For CSA coatings applied to ventilated building façades, the operating temperature does not exceed 450 °C, thereby preventing the formation of the TiAlO_2_ phase and avoiding the severe optical performance degradation associated with this phase transformation. Moreover, under realistic service conditions, the façade surface temperature is typically maintained below 150 °C, implying a long effective service lifetime in practical architectural applications.

Varying the thickness of thin-film layers alters the optical path difference, and hence the phase difference in reflected light, enabling precise tuning of the perceived color and optical performance [16,29,30]. For example, Feiliang Chen et al. [29] reported a Cu/TiN_x_O_y_ /TiO_2_/Si_3_N_4_/SiO_2_ multilayer structure in which a palette of sixteen distinct colors was achieved by adjusting the thickness of the SiO_2_ layer, while maintaining a high solar absorptance exceeding 92% and a low thermal emittance below 5.5%. In addition, Jing Zhang et al. [30] provided a comprehensive review of solar absorbers, systematically summarizing their color-tuning mechanisms and broad application potential across various technological fields.

The XRD analysis was conducted to clarify the origin of the anomalously reduced degradation rate of solar absorptance observed at an annealing temperature of 550 °C, although a higher annealing temperature would, in principle, be expected to cause a more pronounced decrease in solar absorptance. The results indicate that at 550 °C, a chemical reaction occurs at the Al_2_O_3_/TiO_2_ interface, forming a new compound, TiAlO_2_. This interfacial reaction alters the optical path length within the multilayer structure, thereby modifying the solar absorptance.

#### 3.3.4. Corrosion Test

**Figure 9:**
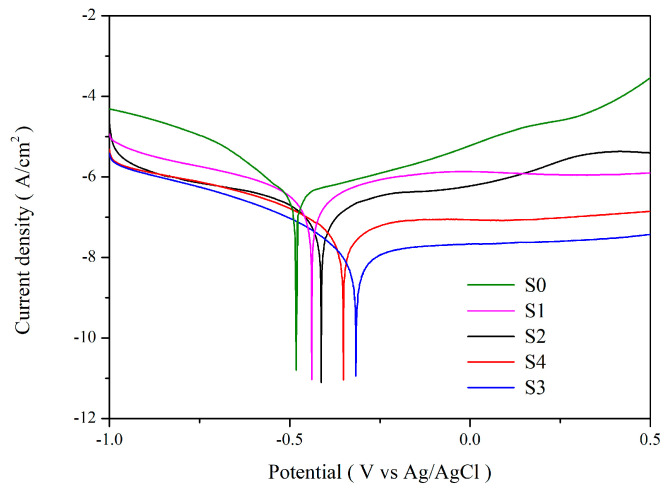
Comparison of the Evans diagrams for uncoated (S0) and CSA-coated (S1–S4: CEP, C#1500, C#1000, and C#600) samples immersed in 1 M NaCl solution.

Figure 9 presents Evans diagrams for the five samples immersed in a 1 M NaCl solution. The samples include: (a) S0 (uncoated mirror-polished SS304 substrate); (b) S1 (CEP coated); (c) S2 (C#1500 coated); (d) S3 (C#1000 coated); and (e) S4 (C#600 coated). The analysis was conducted by extracting the linear Tafel regions from both the anodic and cathodic branches of the polarization curves and extrapolating these regions to their intersection, from which the corrosion potential (ϕ_corr_) and corrosion current density (i_corr_) were determined [5,28]. The corrosion rate (R_corr_) was then calculated from i_corr_ using Faraday’s law. Table 5 summarizes the extracted values of corr, icorr, and Rcorr. SS304 inherently exhibits good corrosion resistance; however, the coated sample S1 shows i_corr_ and R_corr_ values approximately three times lower than those of the uncoated sample S0, indicating a slight enhancement in corrosion resistance after coating. Notably, the i_corr_ and R_corr_ values of S2, S3, and S4 are reduced by nearly one to two orders of magnitude compared with S0. A clear trend is observed: as the surface roughness increases, i_corr_ and R_corr_ decrease, while the ϕ_corr_ shifts toward more positive values. This behavior suggests that the CSA coating is more effective in suppressing the electrochemical activity of the substrate in corrosive environments when deposited on roughened surfaces. These findings are consistent with previously reported results in the literature [28]. The improved corrosion resistance observed for coatings deposited on rough substrates is likely attributable to increased coating thickness and enhanced interfacial adhesion achieved on such surfaces. Among all samples, S3 (C#1000) exhibits the best corrosion resistance, indicating that an appropriately roughened surface is critical for achieving optimal corrosion protection.

### 3.4. Discussion and Summary

This study systematically demonstrates that high-brightness colored solar selective absorber coatings can simultaneously achieve high visible reflectance and high solar absorptance through appropriate multilayer design and surface roughness engineering. Dispelling the myth that “brightly colored CSAs have low solar-thermal performance,” experimental results on reflectance spectra and chromaticity distributions confirm that CSAs with maximum visible reflectance exceeding 15–40% can still maintain α values above 87% and even surpass 93%. These results demonstrate that brightness and α are not mutually exclusive, but can be optimized concurrently through design.

Surface roughness is shown to play a pivotal role in governing both optical and environmental performance. Increasing substrate roughness enhances α by approximately 2% while reducing peak reflectivity by 7–20%, yet without sacrificing the overall high-brightness appearance. More importantly, roughness-induced diffuse scattering effectively mitigates strict specular reflection constraints, significantly broadening the viewing angle range over which the intended color remains visible. Compared with mirror-like substrates, roughened surfaces—particularly the C#1000 condition—preserve color dominance over a wider range of viewing angles under multiple illumination geometries. This improvement arises from the redistribution of reflected light over a wider angular space, reducing abrupt brightness loss and color shift governed by Snell’s law-limited specular reflection.

Beyond optical performance, surface roughness also significantly impacts environmental durability. Vickers indentation tests reveal that roughened substrates promote superior interfacial adhesion and crack resistance through enhanced mechanical interlocking, with the C#1000 sample exhibiting the most robust adhesion behavior. Wettability measurements further indicate that all CSA coatings exhibit hydrophobic characteristics (contact angle > 90°), while moderate roughness maximizes self-cleaning performance. It is worth noting that the rough surfaces of the membranes in this material and structure are all hydrophobic, but rougher is not always better. The C#1000 membrane has the best hydrophobicity.

Thermal stability and lifetime analysis demonstrate that the Al_2_O_3_/TiO_2_–TiON–C multilayer system maintains excellent optical integrity up to 550 °C, with degradation rates below 2%. XPS and XRD analyses reveal that the absorber layer consists of TiO_2_, TiON, and non-crystalline carbon, with no evidence of TiC formation. Annealing at 550 °C induces the formation of TiAlO_2_. Based on the lifetime estimation prediction of the Arrhenius equation, the long-term feasibility of this coating in building applications at 400 K is predicted.

Corrosion testing further confirms that CSA coatings significantly enhance the corrosion resistance of SS304 substrates, particularly when deposited on appropriately roughened surfaces. The observed reduction in corrosion current density by up to two orders of magnitude, accompanied by positive shifts in corrosion potential, indicates effective suppression of electrochemical activity. The superior performance of the C#1000 sample underscores the synergistic effect of optimized roughness, coating thickness, and interfacial adhesion in achieving durable protection.

In summary, this work establishes a comprehensive structure–property–performance relationship for high-brightness CSA coatings. By integrating multilayer optical design with controlled surface roughness, the coatings simultaneously achieve high α, high brightness, enhanced viewing-angle range, and excellent environmental durability. These findings provide clear design guidelines for next-generation colored solar selective absorbers, supporting their practical deployment in energy-efficient building façades where aesthetic flexibility, long-term stability, and thermal efficiency are all critical requirements.

## 4. Valuable Architectural Applications for High-Brightness, High-Solar-Absorptance, and Long-Lifetime CSA Coatings

For a material to be considered truly valuable, it must not only exhibit superior properties but also demonstrate strong feasibility, applicability, and acceptance in practical applications. Therefore, to enhance the practical building applications of high-brightness, high-absorptance, and long-lifetime colored solar absorbers, it is essential to align material performance with effective building construction practices. The perceived color of a CSA is strongly governed by fundamental optical principles, particularly the law of reflection, in which the angle of reflection equals the angle of incidence. When collimated sunlight impinges on a mirror-smooth CSA surface, the reflected light is confined to a narrow angular range; consequently, the CSA appears brightly colored only along the specular direction, whereas at other viewing angles it appears dim or dark. In contrast, when sunlight illuminates a roughened CSA surface, microscale fluctuations in surface-normal vectors induce diffuse reflection, redistributing the reflected light uniformly across a broad range of viewing angles. As a result, both color and brightness remain clear, stable, and angle-independent. Under overcast conditions, although cloud scattering reduces the total incident irradiance, the light arrives from multiple directions. Upon interacting with a rough CSA surface, this multi-directional light is again diffusely scattered, preserving high visibility and stable color appearance from all viewing angles. Such robust angular color stability directly resolves one of the major limitations of conventional dielectric–metal absorber coatings. From the architectural applicability, meeting the expectations of building designers and façade engineers requires CSAs to exhibit a roughened surface morphology, which ensures angle-stable coloration, enhanced visual clarity, and consistent aesthetic performance across diverse lighting environments.

In architectural practical building applications, achieving a perfect color match between CSA coatings and building façades is extremely challenging. To reproduce an exact façade color, the film thickness of each dielectric and absorber layer must be controlled with nanometer-level precision. Such stringent tolerances slow down production and significantly increase fabrication costs, which is why current green buildings typically integrate only black or dark-blue SSAs—colors that are easy to fabricate but visually restrictive. Consequently, façade-integrated SSA technologies remain uncommon in modern architecture. In contrast, when CSAs are intentionally designed to employ strong complementary or high-contrast colors, the requirement for exact color matching with the façade is eliminated. Even slight variations in film thickness do not produce visually noticeable mismatches, enabling rapid fabrication at significantly lower cost. This approach dramatically improves architectural acceptance.

The CSA coatings in Section 4 are essentially the DADA structure and materials from Section 3.

### Highlighting the Architectural Value of CSA Panels

**Figure 10:**
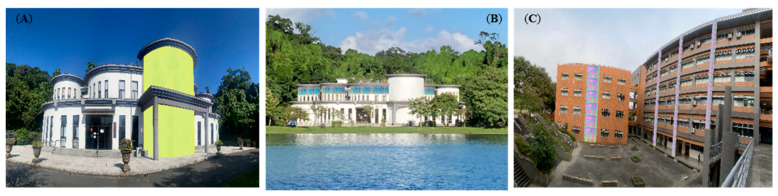
Digital composites of CSA panels in architectural applications. (**A**,**B**) Two design schemes for the Bihu Park Administration Office: (**A**) high-contrast vivid yellow and (**B**) harmonious pastel-blue motifs. (**C**) Patterned CSA panels with a repeating colored grid applied to the Finance Building at NKUST, illustrating the aesthetic potential of high-brightness solar absorbers.

For example, Figure 10 illustrates architectural visualization, demonstrating the design versatility and aesthetic impact of high-brightness, colored solar absorbers. The generation of patterns and colors results from phase differences induced by thickness variations, leading to color shifts (analogous to [29]) These thickness variations are controlled by adjusting the deposition time through shutter masking (i.e., regulating the layer thickness). Figure 10A,B illustrate the CSA panels, fabricated in this study, are digitally composited onto the exterior of the Bihu Park Administration Office in Taipei, Taiwan, demonstrating the strong visual impact achieved when high-brightness CSAs are applied in a contrast-color scheme. As shown in Figure 10A, a vivid yellow CSA applied against a white façade produces a high-contrast visual composition that energizes the overall architectural appearance. In contrast, Figure 10B illustrates a design inspired by the building’s lakeside setting, where gentle breezes generate layered ripples across the blue water surface. By pairing a pastel-blue CSA motif with the adjacent white façade, the resulting composition achieves a harmonious and contemporary aesthetic that reflects both the natural surroundings and the design intent. Figure 10C presents an additional architectural visualization in which a high-brightness CSA—composed of vertically aligned solar-absorber panels patterned with a repeating grid of pastel-colored squares—is digitally mapped onto the façade of the Finance Building at National Kaohsiung University of Science and Technology. These renderings illustrate how vivid coloration can create a dynamic, visually striking exterior aesthetic that enhances both the modernity and recognizability of the building. More importantly, these findings demonstrate that aesthetic integration can be achieved without sacrificing functional performance, indicating that vivid coloration and high solar–thermal efficiency are compatible. This contrast-color design strategy allows CSAs to be manufactured more rapidly and at significantly reduced cost, thereby facilitating large-scale adoption in green building applications. When combined with their high brightness, such CSAs not only enhance the architectural aesthetics of modern structures but also improve environmental performance by delivering high α. Their inherently strong self-cleaning capability further ensures that the panels maintain visual cleanliness for extended periods without frequent maintenance—an important advantage for large building envelopes.

Beyond optical performance and manufacturability, CSAs must also meet long-term sustainability requirements to be viable for real-world deployment. These include strong adhesion, excellent corrosion resistance, and long operational lifetimes. In this study, the CSAs are based on a roughened dielectric/absorber/dielectric/absorber multilayer architecture, in which the absorber layer consists of a TiO_2_–TiON–C composite. This design enables CSAs with Rmax > 20%, α > 0.90, wide viewing angles, strong self-cleaning functionality, and long projected service lifetimes. These attributes collectively demonstrate that the proposed CSAs possess not only high technical performance but also substantial practical applications in building. Their manufacturability, durability, and architectural versatility strongly suggest that they are well-positioned for widespread adoption in next-generation, sustainable, building-integrated solar-thermal systems, especially in green buildings.

## 5. Conclusions

This study demonstrates the feasibility of high-brightness CSA coatings based on a dielectric/absorber/dielectric/absorber multilayer architecture for practical building applications. By integrating optical multilayer design with controlled surface roughness, the proposed coatings achieve stable coloration over a wide range of viewing angles, high solar–thermal absorptance, and long-term environmental durability. Beyond technical performance, these attributes also support a practical marketing strategy, enabling the use of high-contrast and complementary colors that relax strict color-matching requirements, reduce fabrication cost, and enhance architectural acceptance. Collectively, the results confirm the strong potential of D/A/D/A-structured CSAs for large-scale adoption in building-integrated solar–thermal systems.

## Figures and Tables

**Table 1:** Surface conditions, solar absorptance (α), maximum reflectivity (R_max_) in the visible region, and corresponding wavelengths of the specimens shown in Figure 3. ‘CEP’ denotes coatings on electropolished substrates, while ‘C#1500’, ‘C#1000’, and ‘C#600’ represent coatings on electropolished substrates further polished with #1500, #1000, and #600 grit papers, respectively.

Sample	Sample Condition	α (%)	R_max_ in the Color Region (%)	Corresponding Wavelength (nm)
a	CEP	86.1	44.36	510
b	C#1500	88.1	44.09	480
c	CEP	89.4	39.34	450
d	CEP	87.1	21.74	456
e	C#1000	90.4	15.33	679
f	CEP	88.5	26.87	588
g	C#600	90.4	20.87	576
h	CEP	88.4	30.72	515
i	C#1000	90.2	23.72	569
j	CEP	87.2	49.64	487
k	C#1000	91.6	30.09	483
l	CEP	88.0	27.14	604
m	C#1000	90.8	18.49	657
n	CEP	85.9	38.42	443
o	C#1000	92.8	20.62	416

**Table 2:** Vickers micro-indentation results under a 100 g load for the CEP and C#1000 samples.

Surface Condition	Image	Observation and Interpretation	Adhesion Assesment
CEP	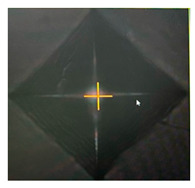	Clear indentation, sharp edges, no cracking or delamination Coating fully withstands stress, strong interfacial adhesion	Excellent
C#1000	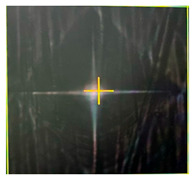	Indentation nearly absorbed, dark uniform surrounding area, no cracks and no delamination Suggests highest toughness and interface strength—coating absorbs deformation	Very strong adhesion (Better adhesion)

**Table 3:** Water contact angles of CSA coatings deposited on substrates with different surface roughness levels (CEP, C#1500, C#1000, and C#600).

Sample	Side-View Image	Water Contact Angle (°)
CEP	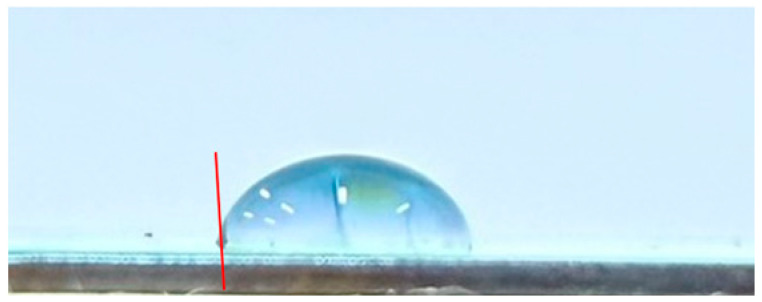	92
C#1500	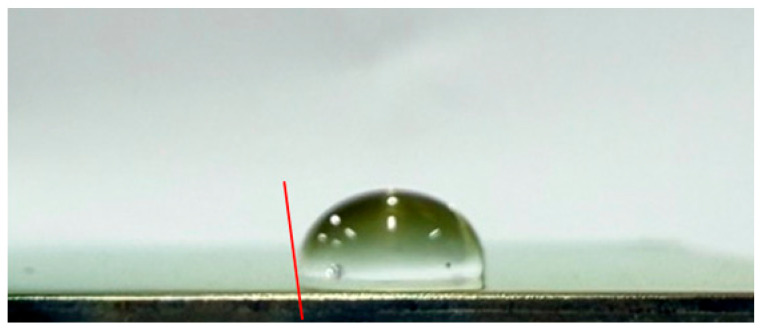	95
C#1000	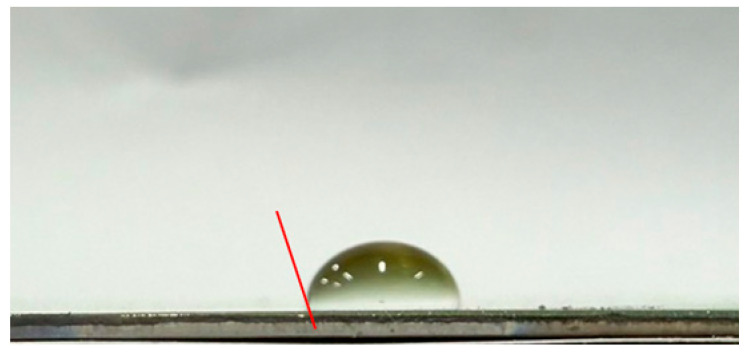	108
C#600	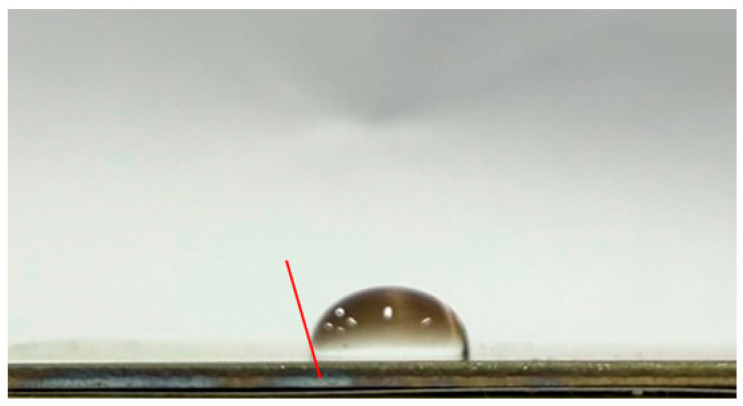	103

**Table 4:** Thermal stability parameters and solar absorptance (α) variation of the specimens shown in Figure 8.

Annealing Temperature (°C)	α Before Annealing (%)	α After Annealing (%)	Degradation Rate (%)
350	91.04	90.13	0.9
450	91.28	89.85	1.57
550	91.11	90.18	1.02

**Table 5:** Potentiodynamic polarization parameters (ϕ_corr_, i_corr_, and R_corr_) for uncoated SS304 (S0) and various coated samples (S1–S4) in 1 M NaCl solution.

Sample	Corrosion Potential ϕ_corr_ (V)	Corrosion Current Density i_corr_ (A/cm^2^)	Corrosion Rate R_corr_ (mm/Year)
Uncoated	−0.48	3.95 × 10^−7^	4.57 × 10^−3^
CEP	−0.44	1.31 × 10^−7^	1.97 × 10^−3^
C#600	−0.41	6.37 × 10^−8^	9.5 × 10^−4^
C#1000	−0.32	5.60 × 10^−9^	6.59 × 10^−5^
C#1500	−0.35	2.53 × 10^−8^	2.98 × 10^−4^

## Data Availability

The original contributions presented in this study are included in the article. Further inquiries can be directed to the corresponding author.

## Abbreviations

CSA	Colored Solar Absorber
D/A/D/A	Dielectric/absorber/dielectric/absorber
α	Solar Absorptance
R_max_	Maximum Reflectance
